# 

**Published:** 1822-09-01

**Authors:** 


### BIBLIOGRAPHICAL RECORD.*

1. The New British Domestic Herbal; or a correct Description of British Medicinal Plants; intended for the use of families, and for every purpose of domestic medicine; illustrated by plates exhibiting one hundred and thirty-two figures of English plants, accurately coloured according to nature. By JOHN AUGUSTINE WALLER, Translator of Orfila, Author of a Treatise on Incubus, &c. Octavo, pp. 408, and 33 plates, Cox and Son, Borough, 1822, price 18*s*. plain, or 1*l*. 10*s* coloured.

---

2. An Essay on Mineral, Animal, and Vegetable Poisons; in which the symptoms, mode of treatment, and tests of each particular poison, with the general morbid appearances on dissection, are concisely detailed: to which is added, an Account of the Means to be employed in Cases of Suspended Animation. Second Edition, duodecimo, pp. 75, with 15 plates. Cox and Son, Borough, 1822, price 3*s*. 6*d*. boards.

☞ *This is in the manner of the toxicological charts, but adapted for the pocket, and likely to prove a popular little vade-mecum of its kind.*

---

3. Cases in Surgery; selected from the records of the Author's practice at the St. George's and St. James's Dispensary; and illustrating the nature and mode of treatment of strumous or scrophulous ophthalmia; the sedative powers of tartar emetic in the cure of local inflammation, when administered internally; the treatment of the mammary or milk abscess; and the beneficial effects of elm-bark, as a cheap substitute for sarsaparilla, with two plates. By HENRY JEFFREYS, Esq. Senior Surgeon to the St. George's and St. James's Dispensary; Assistant Surgeon to the Lock Hospital; and formerly a Surgeon in the 3d Regiment of Foot Guards. Octavo, pp. 237, 8s. bds. London, 1820.

☞ *To be reviewed in our next number.*

---

* Authors and publishers will readily perceive the advantage of having works registered on this List, *with full title pages*, which stand as perpetual advertisements afterwards, the Bibliographical Record forming an integral part of the Journal. Authors are requested to give particular directions to their publishers to transmit works as soon as published, for registry in this department of the Journal, as no works can be inserted that are not transmitted for that purpose.

4. A Sketch of a New Nosological System for the Classification of Diseases. By Robert Tytler, M. D. Octavo, sewed, pp. 14. Calcutta, 1821.

---

5. Observations on some Points relating to the Anatomy, Physiology, and Pathology of the Nervous System. By Joseph Swan, Member of the Royal College of Surgeons, and Surgeon to the Lincoln County Hospital. Octavo, pp. 98, with nine plates. London, 1822.

---

6. Observations on the Use and Abuse of Friction: with some remarks on motion and rest, as applicable to the cure of various surgical diseases. By John Bacot, Member of the Royal College of Surgeons, London. Octavo, sewed, pp. 40. Callow, 1822. Price 2s.

☞ *This little pamphlet contains judicious observations on friction, and very properly discriminates those cases where it may, and where it may not, be advantageous.*

---

7. A Case of an unusually large Aneurism of the Right Axillary Artery, in which the Subclavian Artery was tied. By Charles H. Todd, one of the Senior Surgeons to the Richmond Surgical Hospital. Octavo, p. 13. Dublin, 1822.

☞ *See page 402 of this number.*

---

8. A History of a severe Case of Neuralgia, commonly called Tic Douloureux, occupying the nerves of the right thigh, leg, and foot, successfully treated; with some observations on that complaint, and on its causes, as they vary in different individuals. By G. D. Yeats, M. D. F. R. S. Fellow of the Royal College of Physicians, &c. Octavo, sewed, pp. 55. London, 1822.

---

9. Cases of Neuralgia Spasmodica, commonly termed Tic Douloureux, successfully treated. By Benjamin Hutchinson, Fellow of the Royal College of Surgeons of London, &c. &c. *Second Edition*, pp. 189. London, 1822.

---

10. Considerations sur la Fievre Jaune. Par Le Baron Larrey, &c. &c. Second Edition, octavo. Paris, 1822.

☞ *We return our thanks to the Baron for this little work, and beg to say that we shall take an early opportunity of rectifying our misapprehension of what the Baron meant by "saignées locales," in his Russian Campaign. His message, by Dr. Archer, was received, and the Journal shall be regularly transmitted to him.*

---

11. The Vaccine Scourge: Part II. containing the New Beggar's Opera, &c. Octavo, pp. 106.

☞ *We have been requested to hold up to extensive view the knaves, and the demi-Charlatans, so well delineated in the above pamphlet. But we cannot sully our pages with their names, and as to their characters, they are now pretty well appreciated by society at large. Of these worthies we may say, as Pope said of Dennis:—*

*"On men so poor we cannot take the law;*
*"On men so old we scorn our swords to draw;*
*"Uncaged then let the harmless monsters rage,*
*"Secure in dulness, madness, want, and age."*

---

12. A Case of Hydrothorax, in which is related the origin of the disease, and progress of cure, from the most hopeless state to a return to health: with some observations on Hydrargyri Submurias. By W. Whincopp, M.D. Octavo, sewed, pp. 21. 1822.

☞ *This was a man upwards of sixty, whose constitution was much broken by intemperance, and whose lower extremities had been, for years, œdematous. He was seized with evident symptoms of hydrothorax, and was treated with digitalis at first, and subsequently with digitalis and the sulphate of iron. Great diuresis was the consequence, and a cure was effected, as far as could be expected at his age, and in his constitution.*

---

13. The Principles of Inflammation and Fever. By C. E. Lucas, M. D. Octavo, pp. 304. London, 1822.

---

14. Researches respecting the Medical Powers of Chlorine, particularly in Diseases of the Liver; with an Account of a new Mode of applying this Agent, by which its Influence on the System can be secured. By William Wallace, M. R. I. A. Member of the Royal College of Surgeons in Ireland; one of the surgeons to the Charitable Infirmary, Jervis Street; Surgeon to the Dublin Infirmary for Diseases of the Skin; Lecturer on Anatomy and Surgery, &c. Octavo, pp. 162. London, 1822.

---

15. We received the following printed and manuscript Works in a packet, on the 23d July, from "an individual who is much indebted to Dr. Johnson;" and to this unknown individual we return our sincere thanks. The editor would be glad of an interview, if the individual be in London.

1. Manifesto Acerca de Origen y Propagacion de la calentura que la Reinado en Barcelona, en el anno 1821. Par una Reunion Libre de Medicos Estrangeros y Nacionales. Segunda Edicion, pp. 20. Madrid, 1822.

2. La Medicina Constitucionalizada y Revolucionada por las ciencias exactas o la muerte de los falsos medicos seguida de una carta confidencial, &c. &c. Por D Juan Leymerie, M. D. &c. &c. Octavo, pp. 66. Madrid, 1820.

3. El-Medico Fiscal. &c. *By the same author.* Octavo, pp. 27. Madrid, 1821.

4. Relacion Medico-Politica sobre la Aparicion de la Fiebre Amarilla a ultimos de gulio y Principios de Agosto de 1821, &c. Escrita por El Dr. D. Juan Francisco Bahi, &c. &c. Octavo, pp. 33. Mataro, 1821.

5. A Manuscript Letter from Dr. Francisca Puga, Director of the College of Medicine and Surgery in Cadiz, to James R Mathews, Esq. Dated Cadiz, October, 1819. Foolscap, pp. 14.

☞ *This is a very curious Letter, and contains many sensible reflections on the Cadiz Epidemic, of which the author appears to have seen a great deal. He comes to the conclusion that it affects the same individual but once—that it is personally contagious—and that it is an* internal exanthema, *resembling the external eruptive diseases, but differing in the seat of the eruption, which is the mucous membrane of the stomach and bowels.*

---

16. Analytic Physiology. By Samuel Hood, M. D. A B. Octavo, pp. 200. 1822.

---

17. Tentamen Medicum inaugurale de quinto Nervorum Pari. By John Waldron Watson, M.D. *Ed.* Octavo, pp. 34. Ed. 1822.

☞ *Dr. Watson we have known for some years as a zealous and indefatigable cultivator of anatomical studies. He is now deservedly crowned with the " summis in medecina honoribus ac privilegiis rite et legitime consequendis," in the venerable alma mater of the North. We hope soon to have an opportunity of making some extracts from this very able dissertation on a most intricate subject.*

---

18. Dissertatio Medica inauguralis quædam de Dysenteria Complectens. By Alex. Fraser M'Laughlan, M. D. *Ed.* Octavo, pp. 38. Ed. 1822.

☞ *This is a very well constructed thesis, and we coincide entirely with Dr. M'Laughlan in the views which he takes of the etiology, pathology, and treatment of the disease. We shall quote a short etiological extract as a specimen.*

" *Quamvis sine dubio, Dysenteriæ intestinorum et inflammatio et strictura plerumque insint, attamen, ni fallor, de horum causa primaria inter pathologos adhuc agitur : quoad hoc, sentio auctorem recentem, qui perspirationem restrictam morbi causam primariam proximam existimat, rem rectè tenuisse. In Dysenteriâ igitur, vasis externis superficiei cutis corrugatis, perspiratio cutanea coercitur ; quod testantur omnia symptomata initio morbi conspicua, viz. horrores quibus succedunt magna sitis, arida contractaque cutis. Extremis vasis sic constrictis, circuitus equilibrium amittitur ; sanguis ab externis ad internas partes transit ; inde, in regionibus calidis præsertim, oritur magna vasorum mesenteriæ ac venarum portarum plethora. Excretione naturali per vasa cutanea sublatâ, intestina vicario munere fungi oportet ; inde mucus, vias naturaliter protegens, majore copiâ justo secernitur. Eodem tempore a magna plethora internarum partium intestinorum sæpeque jecoris inflammatio incipit.*

" *In intestinis major vis sanguinis, ad mucum secernendum, ministratur ; et æger dum dejicit vas sanguinem vehens sæpe perrumpit ; morbo tamen progrediente, ac plethorâ auctâ, vasa apud partes internas sanguine adeo turgida fiunt, ut ultimo perrumpantur ; hoc modo excretiones sanguineæ omnino videntur.*"

---

19. Observations, from Experience, on the Aid obtained in various Diseases, particularly those incidental to Tropical Climates, by the external Application of the Nitro-Muriatic Acid in a Bath ; together with several Cases in which it has been used by the Author with great efficacy ; to which is added, the present most approved mode of mixing the Acids and preparing the Bath. By Phineas Coyne, Member of the Royal College of Surgeons of London; and late of the Honourable East India Company's Service. One vol. 8vo, pp. 140. London, 1822.

---

20. The Study of Medicine; comprising its Physiology, Pathology, and Practice. By John Mason Good, M. D. F. R. S. &c. Member of the Royal College of Physicians of London. Four volumes, 8vo. London, August 1822.

---

21. On the Use of the Moxa, as a Therapeutical Agent. By Baron D. J. Larrey, Surgeon in Chief to the Hopital de la Garde Royale, &c. &c. &c. Translated from the French, with Notes, and an Introduction, containing a history of the substance. By Robert Dunglison, Fellow of the Royal College of Surgeons, &c. &c. Octavo, pp. 148. London, 1822.

---

22. An Essay on the Epidemic Cholera of India. By Reginald Orton, Assistant Surgeon in His Majeety's 34th Regiment of Foot. Vol. the First, pp. 421. Madras, 1820.

---

### IN THE PRESS.

No. I. of Anatomical and Physiological Commentaries. By Herbert Mayo, Surgeon and Lecturer on Anatomy.

Mr. SHAW has in the Press a Work on Distortions.

The First Part will treat of the Distortions of the Trunk to which young persons are subject, in consequence of habitual bad posture, and the neglect of proper exercise. Their varieties will be illustrated by engravings of distorted Skeletons, and sketches will be given of the postures and modes of Exercise, and of the application of Instruments, calculated to correct each deformity.

The Second Part will treat of the Scrophulous Diseases of the Spine. To this will be added an account of the extensive collection of Specimens of Distortions from various causes preserved in the Anatomical Museum, Great Windmill Street.

The "Anatomie Generale" of Bichât, translated from the French, is now in the Press, and publishing by Subscription, in Four Volumes, price Thirty Shillings.

### *Additional Subscribers since last Quarter.*

A

Autenrieth, Dr. (Jun.) Tubingen

Abercromby, Dr. John, Edinburgh

Abbott, Mr. Robert, Surgeon, Needham Market

Association (Medical) of the Royal College of Pysicians of Ireland

B

Bell, Mr. Staff Surgeon, Sligo

Browne, Mr. Tobias, Surgeon, Camberwell

Bacot, John, Esq. London

Bell, Mr. James, Surgeon, Cockermouth, Cumberland

Belcher, William, M.D. Licentiate of the Royal College of Surgeons of Ireland, Bandon

Brown, Mr. R. Member of the Royal College of Surgeons, London, Preston, Lancashire

Bow, Wm. Forrester, M. D. Physician to the Alnwick Dispensary

Boxwell, Surgeon, Abbyleix

Byron, Luke, Esq. M. D. Kells, Ireland

C

Conry, Dr. Dennis, St. Petersburgh

Carter, Dr. Sligo

Caldwell, Mr. Surgeon, Milbrook, Southampton

Craigie, Dr. Bengal Establishment

Cannam, Mr. Surgeon, Stevenage, Herts

Cheyne, John, M. D. Physician General, &c. Dublin

Colles, Ab. Esq. M. D. Professor, Royal College of Surgeons, Ireland

Clarke, Joseph, Esq. M. D. Dublin

Calendar, J. Esq. 7th Dragoons

D

Dickson, Mr. John, Surgeon, Rio Janeiro

Diehl, Dr. Three Rivers, Lower Canada

Dent, Dr. Member of the Royal College of Physicians, and Physician to the Stafford Infirmary

De Renzy, Dr. Carnew

Desmond, John, Esq. M. D. Youghal

Dempster, Dr. Roscrea, Ireland

Dunn, Dr. Drumsna, Ireland

Duggan, James, Esq. M. D. Dublin

Daunt, Henry, Esq. Dublin

F

Francke, Dr. Stutgard

Falloon, Daniel, Esq. M. D. Dublin

G

Glasgow University Medical Library

Greenock Medico-Chirurgical Association

Garrett, Mr. G. Surgeon, Kington, Herefordshire

Groat, Dr. Hart Street, Edinburgh

Gardner, Dr. Eden, Edinburgh

H

Hopfengartner, Dr. Physician, Stutgard

Hasper, Dr. Stutgard

Hill, N. Esq. M.D. Greenock

Headley, Mr. Surg. Cambridge
Hannah, Mr. Surgeon, Alford
Holt, Dr. East Hill, Colchester
Hemphill, Surgeon, Cashel, Ireland
Hunter, Dr. Dublin Castle

I & J

Inman, Mr. Surgeon, Strand
Jeffreys, Mr. Julius Assistant Surgeon, Calcutta
Johnson, Andrew, Esq. Professor, Royal College of Surgeons of Ireland
Jones, Mr. Surgeon, Strand

K

Kelly, Dr. Leith
Kennedy, George, Esq. M. D. Dublin

L

Love, Mr. John, Assist. Surg. H. M. S. Queen Charlotte, Portsmouth
Larrey, M. Le Baron, Paris
Litchfield, Mr. Surg. Keppel Street, Russel Square
Lleigh, Mr. R. P. Surg. Hull

M

M'Laughlan, Dr. Alexander, Fraser, Edinburgh
Marsh, H. Esq. M. D. Molesworth Street, Dublin
Mills, T. Esq. M. D. Dublin

O

O'Beirne, James, M. D. Surg. in Ordinary to the King

P

Pratt, Mr. Chemist, King Street, Edgeware Road
Pope, Dr. (place not stated)
Peebles, John, Esq. Dublin

R

Royal College of Surgeons, Ireland
Richmond Physical Association, Dublin

S

Smith, Dr. Robt. (erroneously stated John) Maidstone
Salemi, Dr.
Sutherland, Mr. Robert, Member of the Royal College of Surgeons of London, Assist. Surgeon, Madras Establishment
Smith, Charles, Esq. Upton-upon-Severn
Scott, Francis, Esq. Armagh
Sullivan, James, Esq. M. D. Dublin
Speer, Doctor, Dispensary, Castlenock, Ireland

T

Turner, William, Esq. Surg. R. N. Greenock
Thomas, L. Esq. Zante

W

Weir, Mr. Daniel, Bookseller, Greenock, 4 copies
Williams, Mr. Surgeon, His Majesty's Ship Genoa, Sheerness
Wentworth, Mr. Surgeon, Cambridge
Watson, Mr. Surgeon, Berners Street
Wells, Dr. New York
Wright, Dr. (place not stated)
Watson, Dr. John, Waldron
Wise, Mr. Surgeon, Bambury
Winton, Dr (place not stated)
Wright, Mrs. Matron of the Westminster Lying-in Hospital
Wilmer, Mr. Henry, Surgeon, Baker Street
Walker, Dr. Huddersfield

☞ *Subscribers are to procure the Journal through the medium of their own Booksellers, in the same way as they would any other periodical publication.*

